# Anesthestic management of Kassabach-Meritt Phenomenon in an adult: case report

**DOI:** 10.1186/s12871-016-0278-y

**Published:** 2016-11-09

**Authors:** Abdelghafour Elkoundi, Mehdi Samali, Noureddine Kartite, Mohammed Tbouda, Mustapha Bensghir, Charki Haimeur

**Affiliations:** 1 Department of Anesthesiology and Intensive Care, Military Hospital Mohamed V of Rabat, Faculty of Medicine and Pharmacy of Rabat, University Mohamed V-Souissi, Rabat, Morocco; 2 Department of Anatomopatholgy, Military Hospital Mohamed V of Rabat, Faculty of Medicine and Pharmacy of Rabat, University Mohamed V-Souissi, Rabat, Morocco

**Keywords:** Anesthesia, Adult-onset, Kaposiform hemangiendothelioma, Kasabach-Merritt phenomenon, Case report

## Abstract

**Background:**

Kasabach-Merritt phenomenon (KMP) is characterized by a vascular tumor with profound thrombocytopenia and consumptive coagulopathy that may presents significant challenges for anesthesiologist.

**Case presentation:**

An 87-year-old man presented with kaposiform hemangioendothelioma involving the right leg in critical condition due to massive bleeding. Hematology investigations indicated the presence of KMP. Association of this type of tumor with KMP in adults has never been reported.

**Conclusion:**

The present case report lays an emphasis on the potential difficulties during anesthetic management of this rare condition.

## Background

Kasabach-Merritt phenomenon (KMP) is characterized by giant hemangiomas, severe thrombocytopenia and consumptive coagulopathy [1] that may cause life-threatening complications and require aggressive treatment. The anesthetic management of patients with this trouble is challenging and rarely reported [2–4].

We report the anesthetic considerations in what we believe to be the first report of adult-onset KHE associated with KMP.

## Case presentation

An 87-year-old male, weighing 50 kg, presented at emergency department in shock with cool clammy extremities, palor, blood pressure (BP) of 85/35 mmHg and tachycardia with heart rate (HR) of 130.

The past medical history was consistent of hypertension, diabetes mellitus and transurethral resection of prostate under spinal anesthesia. He was on treatment with amlodipine, metformine and dietary restriction. Twelve months ago, he was diagnosed with KHE involving the right leg. A complete excision was performed on the mass and a skin graft was applied from the homolateral thigh. The surgical team successfully followed up the patient for 9 months without recidivism. There were no history of thrombocytopenia or other bleeding diathesis. The patient had not previously undergone any chemotherapeutic agent known to cause cardiotoxicity.

**Fig. 1:**
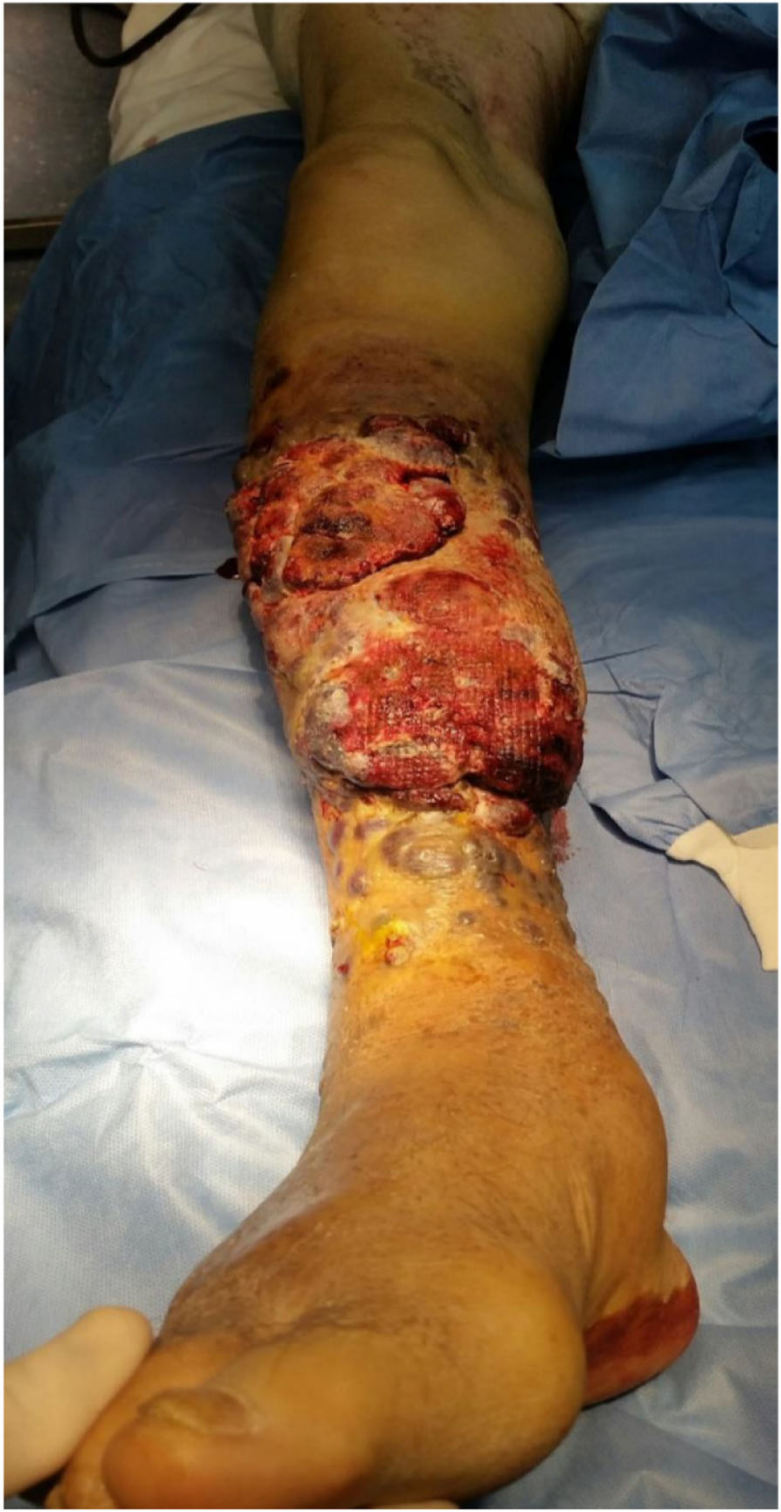
Anterior view of the tumor showing multiple nodules red-purple and bleeding spontaneously with ecchymosis over and around

**Fig. 2:**
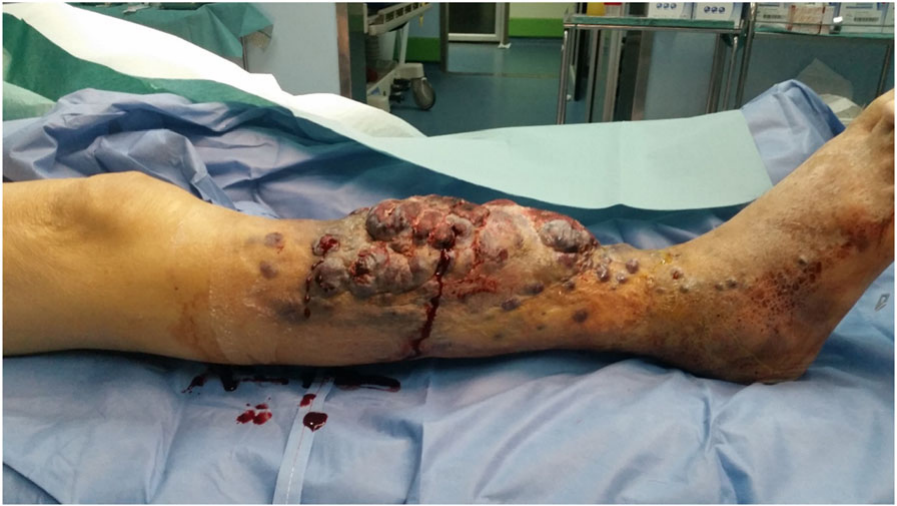
Lateral view of the tumor

On further examination, the patient was afebrile, alert with oxygen saturation of 97 % and respiratory rate of 22. The cardiopulmonary auscultation was normal. The abdomen was soft and free from tenderness on pressure. It was noted the presence of multiple nodules developed in the previous surgical site (Figs. 1 and 2). The tumor mass was painless, immobile and sizing 12 × 6 × 5 cm. The overlying skin was deep red-purple and bleeding spontaneously with ecchymosis over and around the tumor.

Laboratory results demonstrated platelet count 24.000/mm3, normocytic normochromic anemia with hemoglobin 6.3 g/dl, hematocrit 26 %, ASAT 20 IU/L, ALAT 23 IU/L, ALP 70 IU/L, GGT 49 IU/L. Renal function, electrolytes and ABG were normal. The coagulation tests were: prothrombin time of 12.9 s (11 to 13.5 s), plasma fibrinogen concentration of 2.70 g/l ((normal 1.50–3.87 g/L)), and moderate increase in D-dimer 1.2 μg/mL (<0.5 μg/mL).

Laboratory workup revealed no evidence of hemolysis: Bilirubin 0.8 mg/dl (normal 0,3 et 1,9 mg/dL), LDH 232 IU/L (normal 190–390 IU/L). No evidence of anti-immune process was detected to account for destruction of these platelets as direct coombs test was negative. The results of the initial septic screen were unremarkable: C reactive protein of 7 mg/L, White cells count of 4.1 × 10^9^/L and neutrophils of 3.2 × 10^9^/L. Procalcitonin was 0.1 ng/mL (<0.5 ng/mL). Blood and urine cultures at the time of admission demonstrated no bacterial growth.

Heart failure secondary to massive arteriovenous shunt across the lesion was excluded by a normal transthoracic electrocardiography. The chest X-ray demonstrated multiple calcific densities throughout both lungs consistent with calcified haemangiomas and already present in previous radiographies.

Bleeding was not controlled despite compression dressing over the lesion and the transfusion of packed RBCs (04 units) and platelet concentrates (10 units). Methylprednisolone was injected intravenously (5 mg/kg) but no further improvement in platelets or hemoglobin was observed. The case has been discussed in a multidisciplinary setting including hematologist, plastic surgeon and anesthesiologist. In the absence of other alternatives, it was decided to carry out another surgery in order to perform an amputation of the right leg.

Pre-anesthetic evaluation did not reveal any abnormal findings; particularly we excluded any associated hemangioma involving the upper airway.

On arrival in our operating room (<3 h from admission), he had BP of 80/40 mmHg with HR of 125. 3-lead ECG and pulse oximetry were monitored. A 16-Gauge peripheral intravenous line was placed in his right forearm and kept open with isotonic saline solution (2000 ml). A radial arterial line was placed for continuous monitoring of arterial blood pressure and for ABG analysis and serum electrolytes during operation. A double lumen catheter was inserted into the right internal jugular vein under ultrasound guidance and was used as an infusion route for noradrenalin. A rate of 1 μg/kg/min was necessary to reach and maintain a mean arterial blood pressure above 65 mmHg.

**Fig. 3:**
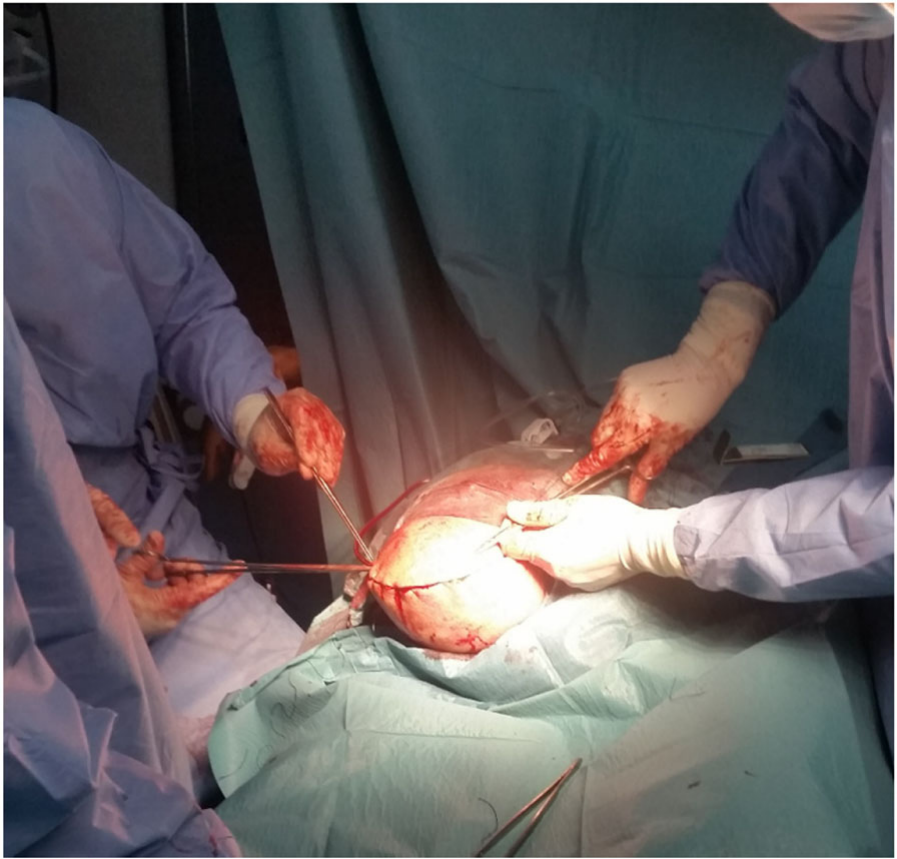
Transfemoral amputation in our patient

After stabilizing the patient and adequate pre-oxygenation of 5 min, general anesthesia was induced with rapid sequence induction using ketamine and succinylcholine. An endotracheal tube size 7.0 mm was easily inserted. The lungs were ventilated with a mixture of oxygen-air (50–50 %) and anesthesia was maintained with 1 vol% of sevoflurane. End expiratory CO2 and inspiratory O2 concentration were monitored. We requested the surgeon to elevate the lower limb and rapidly apply a lower extremity tourniquet in an attempt to minimize blood loss. A transfemoral amputation was made (Fig. 3). As result of the sustained severe hemorrhage in this patient, he required a massive transfusion of blood products which starts earlier concomitant with the tourniquet manoeuver. He received per-operatively a total of 07 units of RBCs, 10 units of platelets concentrate and 04 units of fresh frozen plasma within 2 h. He was also given 4 g intravenous calcium gluconate and 40 mg of furosemide. Pre-warming of blood products and administered fluids and using of patient warming device to prevent hypothermia was done. After the above treatments, the vasopressor support was withdrawn at the end of the surgery. The patient remained hemodynamically stable and had adequate urine output.

**Fig. 4:**
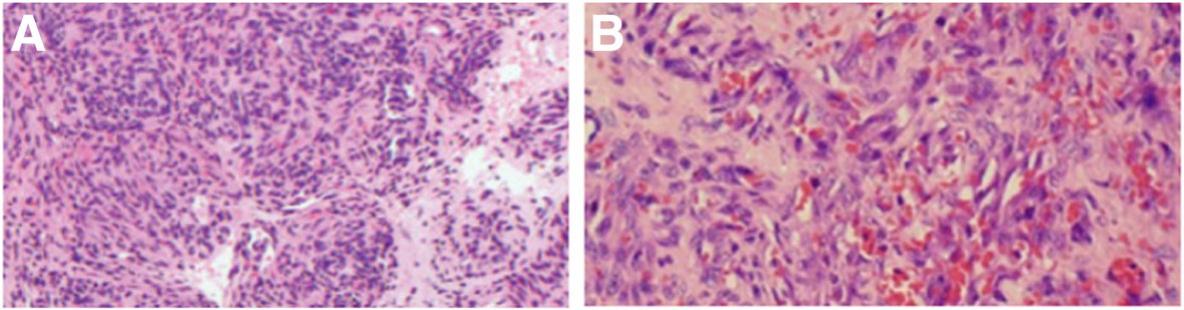
Histological features of the surgical piece. **a** Low power view of Kaposiform hemangioendothelioma shows Clustering of capillaries and spindle cell nodules in larger nodular formations separated by fibrous septa. (hematoxylin and eosin stain, ×200). **b** Higher magnification showing Crescent-like vascular slits and containing RBC’s. (hematoxylin and eosin stain,×400)

Post operatively, he was shifted to the intensive care unit (ICU) with mechanical ventilation support in stable condition. Platelet count rapidly had increased to 66.000/mm3 and hemoglobin had improved to 10,3 g/dl. The patient did not develop complications of massive transfusion and no bleeding of incisional wound was observed. Blood gas analysis showed pH 7.36, PCO 35 mmHg, PaO2 145 mmHg, bicarbonates 22.3 mmol/l. Serum electrolytes were within normal range. Recovery from anesthesia was smooth and quite well and he was extubated in the same day. In the second post-operative day, his report showed platelet count of 115.000/mm3 and hemoglobin of 9.9 g/dl. Anatomopathological examination of the surgical piece has revealed KHE (Fig. 4). The patient was transferred to the department of plastic surgery where the follow-up was uneventful. He was discharged home after 1 month.

## Discussion

KHE is a rare vascular proliferation of intermediate malignancy and can arise during adulthood. The association with thrombocytopenia or consumptive coagulopathy is called Kasabach-Merritt phenomenon (KMP), also known as Kasabach-Merritt syndrome [1]. Although the pathogenesis of thrombocytopenia is not completely understood, it is generally presumed that platelet-trapping by abnormally proliferating endothelium within the hemangioma and secondary activation of the coagulation cascade are factors that lead to this condition [5]. In addition, the platelet half-life is drastically shortened to between 1 and 24 h.

Croteau and colleagues reported that superficial KHE, that are present in older aged people are associated with decreased frequency of KMP [6]. Based on our knowledge, this particular case represents the first report of adult-onset of KMP associated with KHE. The mortality rate of KMP remains high, ranging from 12 to 24 % due to hemorrhage, infection, invasion of vital structures, and multiple organ failure [7, 8]. The high morbidity and mortality rates observed in these patients necessitate aggressive therapeutic intervention [9] of which surgical excision of the tumor appears to be the most effective. Other reported treatments include high-dose systemic corticosteroids, vincristine, interferon, radiotherapy, and propranolol. More recent and promising treatment is sirolimus [10, 11]. Sirolimus has been described to decrease tumor burden by suppressing mTOR (Mammalian target of rapamycin) signaling pathway and causing cell apoptosis. Because of the imminently life-threatening tumor, we estimated that the time to respond to these medical therapies is considered too long and consequently they were not an option. In addition, the effectiveness of these treatments has been inconsistent and currently, there are no known treatment guidelines. Transarterial embolization is an effective therapeutic option specially in case of unresectable tumor due to its large size and infiltrating nature [12]. Unfortunately, this option was not available in our hospital at the time of admission.

Case reports regarding anesthesia in patients with KMP are extremely rare [2–4]. These patients present various challenges to anesthesiologist including: 1) uncontrollable hemorrhage, 2) enlarged lesion, 3) airway obstruction (in cases where the lesion involves the head and neck), 4) heart failure in case where the massive arteriovenous shunt is across the lesion and 5) complications from massive blood transfusions.

The most common complication associated with the anesthetic management of patients with KMP is severe bleeding that can result from the disturbance of blood coagulation. In addition to the severe, persistent thrombocytopenia characteristic of KMP, patients often manifest elevated D-dimer and low fibrinogen [7–13]. Severe anemia is also usually present [5].

A thorough hematologic examination that includes complete blood count, coagulation screening and D-dimer concentration is therefore critical in advance of the surgery.

Platelet transfusion prior to surgery may exacerbate KMP by enlarging the tumor and increasing the pain and should therefore be avoided. Furthermore, de-granulating platelets release proangiogenic molecules and may stimulate the tumor and increase the consumptive coagulopathy [14]. When feasible, the use of tourniquet manoeuver may decrease intraoperative blood loss. Use of antifibrinolytic and antiplatelet agents has had mixed results [8–15]. Replacement of depleted clotting factors using fresh-frozen plasma has been recommended in patients with KMP before surgery [5]. Marked hypofibrinogenemia may necessitate the administration of cryoprecipitate to correct the underlying coagulopathy [16]. Recombinant factor VII therapy was also reported in a child with KMP undergoing major surgery [17].

Another anesthetic problem arises from the location and size of the hemangioma. Kawahara and colleagues [2] paid attention to this point. They reported a case of a 20-month-old girl with KMP who underwent the cryosurgical procedure to remove the angioma from her face. Maintaining patient airways, ventilation and intubation are major anesthetic concerns when the patient’s lesions are located on the face because of challenges such as: 1) fitting an anesthetic mask satisfactorily, 2) bleeding during the endotracheal intubation and 3) airway obstruction resulting from excitement or postural changes. The air-passages should be examined carefully for any associated hemangioma and the patient needs to be sufficiently premedicated to avoid excitement before inducing anesthesia.

The use of a neuraxial anesthetic technique is not a good choice in this case, because of bleeding tendencies and the possibility of associated hemangiomas within the soft tissue or spinal canal.

The use of modern volatile anesthetics reduce the systemic vascular resistance and arterial blood pressure. The intravenous anesthetics that affect the cardiovascular system exert a similar effect. Volatile anesthetics and propofol induce redistribution of blood from the systemic circulation to the venous malformation and can lead to unexpected and disastrous effects in patients with such vascular abnormalities. The combination of both of these agents can have a synergistic effect [18]. In our case, sevoflurane was used in a lower dose (1 vol %) for inhalational maintenance. The use of ketamine seems to be a beneficial alternative to propofol and volatile agents [19].

Additional doses should be administered before and during anesthesia if patients are already treated with steroids to compensate for possible adrenal insufficiency.

Endovascular treatment is very challenging given the complexity and individuality of the vasculature of the tumor. The risks and benefits must be clearly explained to the patient. General anesthesia is generally recommended. Pretreatment with steroids is preferred to control inflammatory response [20]. The procedure should be performed as fast as possible to reduce the catheterization time by practitioners with appropriate training and support. Laboratory studies are performed to test clotting parameters and renal function. Hydration is recommended before the procedure to protect the kidney from the effects of hemolysis [21]. Antibiotics are usually given for 10 days after the procedure due to the risk of infection. Antiemetics and analgesics may also be administered. Postembolization syndrome can also occur and is treated symptomatically [22].

Since massive bleeding may also occur, one should anticipate the onset of hypotension and shock. It is reasonable to invasively monitor these patients. A combination of measures, including: 1) control of surgical bleeding, 2) supportive treatment with rapid fluid resuscitation, 3) massive transfusion of blood products and administration of intravenous vasoactive agents for maintaining tissue perfusion and 4) oxygenation are strongly recommended in patients with severe bleeding. Massive arteriovenous shunt across the lesion can precipitate high output congestive heart failure and the scenario can become more challenging. The anesthesiologist must be aware for signs of heart failure.

Patients with KMP are likely to require massive blood transfusions as illustrated in our case. This condition can be associated with many complications, such as acidosis, hypothermia, dilutional coagulopathy, electrolyte abnormalities, citrate toxicity, and transfusion related acute lung injury (TRALI). None of these complications was observed in our case. Particular attention must be paid to the prevention of secondary coagulopathy due to hypothermia and acidosis. In our case, the patient was prevented from hypothermia by pre-warming all administered fluids and blood products as well as using patient warming devices, and increasing the operating room temperature. The ABG analysis was repeated during the procedure and did not show evidence of acidosis.

In the post‑operative period, patients should be transferred to the ICU with mechanical ventilator support and the extubation must be guided by repeated monitoring of ABG and hematological tests. In the case described here, several different attempts to control the coagulopathy, bleeding and anemia were fruitless and there was no other option than to resection the hemangioma by amputation of the limb as a final option to save the patient. Removal of the hemangioma associated with prompt correction of the patient’s physiologic disturbances leads to a rapid recovery of the hematological profile.

## Conclusion

KMP is a life-threatening condition requiring prompt and effective treatment. The anesthesiologist should be aware of the importance of some key considerations in the anesthetic management of this rare situation and anticipate complications that can lead to severe hemorrhaging, enlarged lesions, airway obstruction, massive transfusion and heart failure.

## Abbreviations

ABG: Arterial blood gas
BP: Blood pressure
HR: Heart rate
ICU: Intensive care unit
KHE: Kaposiform Hemangioendothelioma
KMP: Kasabach-Merritt phenomenon
RBCs: Red blood cells
